# Association of Fall Risk Scores With 30-Day Return to Hospital Rates in Older Adults Following a Hip Fracture: A Retrospective Single-Center Analysis of a Quality Improvement Database

**DOI:** 10.7759/cureus.95818

**Published:** 2025-10-31

**Authors:** Antonio Da Costa, Gabriella Engstrom, Joseph Ouslander

**Affiliations:** 1 Department of Medicine, Florida Atlantic University Charles E. Schmidt College of Medicine, Boca Raton, USA

**Keywords:** 30-day readmission, fall risk assessment, geriatrics, hip fracture, hospital returns, johns hopkins fall tool, older adults, quality improvement

## Abstract

Introduction: Higher fall risk scores have been associated with increased morbidity among older adults. Older adults, particularly after a hip fracture, are vulnerable to hospital returns within 30 days post discharge. The objective of this quality improvement project was to examine whether higher fall risk scores, as measured by the Johns Hopkins Fall Risk Assessment Tool (JHFRAT), are associated with 30-day hospital return rates in adults aged 75 and older hospitalized with a hip fracture. Additionally, an exploratory analysis compared return rates between high fall risk admissions with and without hip fractures to contextualize findings.

Methods: This secondary analysis of a quality improvement database included 61,193 admissions of people aged 75 years and older to a 400-bed community teaching hospital between 2017 and 2020. Admissions were excluded if they died during hospitalization or were discharged to hospice care. Fall risk scores were calculated by hospital nursing staff using the JHFRAT and categorized into low (≤16.0) vs. high (>16.0) risk groups based on previous research. The outcome measure included hospital readmissions, observation stays, and emergency department (ED) visits without hospital admission within 30 days of initial discharge. Chi-square tests were used for statistical analysis, with a significance level set at a two-sided p-value < .05.

Results: Among the 2,539 eligible admissions with a diagnosis of hip fracture, 2,077 (81.8%) were categorized as low fall risk and 462 (18.2%) as high fall risk. There was no significant difference in 30-day return rates between these groups (21% vs. 18%, p = .128). Similarly, among admissions without a hip fracture, return rates did not differ significantly by fall risk category (21% for low-risk vs. 22% for high-risk, p = .107). However, when examining only admissions with high fall risk scores, those without a hip fracture had a significantly higher return rate than those with a hip fracture (22% vs. 18%, p = .02).

Conclusion: High fall risk scores on the JHFRAT were not significantly associated with 30-day returns to the hospital among admissions aged 75 and older with a diagnosis of hip fracture in the hospital we studied. Limitations include the single-center design, potential variability in fall risk scoring due to nurse training differences, and a lack of information on social and post-discharge factors. Additional analyses in larger, multicenter, and more diverse populations are needed to explore additional factors that are associated with hospital returns in this population.

## Introduction

Falls are a major public health concern among older adults, representing one of the leading causes of injury-related morbidity and mortality worldwide. Approximately one in four individuals aged 65 and older experiences a fall each year [1]. Hip fractures are among the most severe fall-related injuries, frequently resulting in immobility, functional decline, and increased mortality [2,3]. Following a hip fracture, only one-third of older adults recover their prior functional status, and up to 30% return to the hospital within 30 days of discharge [4,5]. These hospital returns can cause additional hospital-acquired complications and costs, and hospitals may be financially penalized for high rates of 30-day readmissions [6]. 

Fall risk assessment tools are widely used in the clinical management of older adults, helping healthcare providers to identify individuals at heightened risk for falls and related injuries and proactively implement preventive measures. Among the most widely used assessment tools in the hospital setting is the Johns Hopkins Fall Risk Assessment Tool (JHFRAT), which has been validated for predicting in-hospital falls [7]. However, its potential association with broader post-discharge outcomes, such as 30-day return to hospital, remains unclear [8].

Some prior studies suggest that fall risk scores may be associated with unplanned returns to the hospital. For example, Ravi et al. (2017) found that an increased Hendrich Fall Risk Score at discharge was strongly associated with unplanned readmissions following total joint arthroplasty [9]. Similarly, Manemann et al. (2018) found that higher fall risk scores, as measured by the Hendrich II Fall Risk Model, were significantly associated with increased 30-day readmission rates in patients with cardiovascular disease [10]. However, these findings may not generalize to patients hospitalized for acute injuries like hip fractures.

Despite these findings, it remains uncertain whether fall risk scores obtained using the JHFRAT are associated with 30-day hospital readmissions among adults aged 75 and older hospitalized with hip fractures. Although the JHFRAT was developed to identify in-hospital fall risk, it incorporates factors such as mobility, cognition, sensory deficits, and polypharmacy, domains that plausibly extend to broader post-discharge vulnerability [8]. Given that 30-day returns often reflect multifactorial risk, we hypothesized that these intrinsic factors may relate to hospital return risk.

The primary objective of this study was to evaluate whether high fall risk scores, as measured by the JHFRAT, are associated with increased rates of 30-day return to the hospital in older adults hospitalized with hip fracture. A secondary, exploratory analysis compared return rates between high fall risk patients with and without hip fracture to contextualize the primary findings and assess whether observed patterns were specific to hip fracture or generalizable to other high-risk older adults.

## Materials and methods

Study design and setting

This was a retrospective, secondary analysis of data from a 400-bed community teaching hospital from 2017 to 2020. The Institutional Review Board (IRB) of Florida Atlantic University, Boca Raton, FL, approved the development of this database as a quality improvement project and granted a waiver of informed consent (approval number: 776896-1). Patients were identified using International Classification of Diseases, 10^th^ Revision (ICD-10) codes for hip fractures (Appendix A) [11]. The dataset was derived from structured fields in the electronic medical record, with fall risk scores documented by nursing staff. Data completeness exceeded 95%.

Patient population

Adults aged 75 years and older admitted to the hospital with a diagnosis of hip fracture (defined using ICD-10 codes [11]) were eligible for inclusion. Each hospital admission was analyzed independently, regardless of prior hospitalizations. Admissions were excluded if fall risk scores were not documented at admission, if the patient died during hospitalization, or if they were discharged to hospice care. Each admission was treated as an independent observation. Approximately 20% of hip fracture admissions were excluded due to missing fall risk scores.

Measurements

Fall risk scores were calculated by hospital nursing staff using the JHFRAT, which has been validated for in-hospital use [7,12]. Based on the validation study, which demonstrated that patients who experienced falls had average scores of 16.3 [12]. We classified admissions into two categories: 1) high fall risk: scores greater than 16, indicating significant impairments in mobility, sensory, or cognitive function that contribute to a higher likelihood of falls; and 2) low fall risk: scores of 16 or below. The >16 threshold has been validated for predicting in-hospital falls but has not been studied in relation to post-discharge outcomes. Its use in this study is exploratory and intended to assess whether intrinsic fall risk domains may relate to early return to hospital [12]. Additionally, characteristics of admissions, such as age, sex, comorbidities (e.g., congestive heart failure, chronic kidney disease, chronic pulmonary disease, and diabetes), and other relevant clinical variables were documented.

Outcomes

The primary outcome was a 30-day return to the hospital, defined as inpatient admissions, observation stays, and emergency department (ED) visits without admission.

Statistical analysis

Chi-square tests were used for comparisons of categorical variables when all expected cell counts were ≥5. Fisher’s exact test was applied when any expected cell count was <5. These tests were used to examine associations between fall risk categories and 30-day hospital return, as well as between clinical characteristics and fall risk status within and across hip fracture groups. No multivariable modeling was performed; this analysis was descriptive and exploratory. Statistical analysis was completed using the IBM SPSS Statistics software, version 29.0 (IBM Corporation, Armonk, NY, USA).

## Results

**Figure 1:**
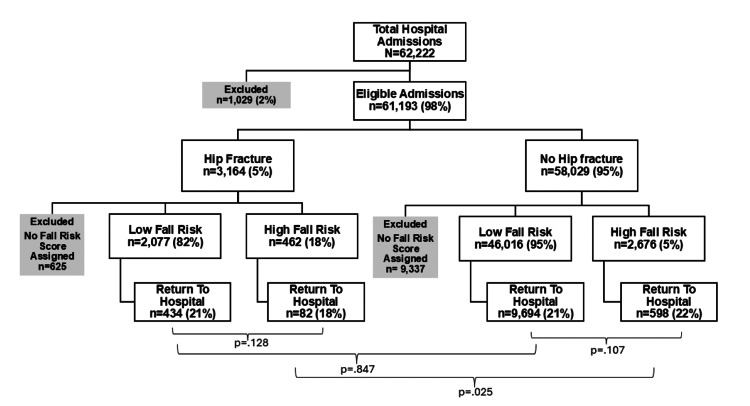
Demographics of Eligible Admissions

Figure 1 illustrates the flow of admissions included in the analysis. Of 61,193 eligible admissions aged 75 years and older between 2017 and 2020, 3,164 (5%) had a diagnosis of hip fracture. After excluding 625 (19.7%) admissions with missing fall risk scores, 2,539 remained eligible for analysis. Among the 58,029 admissions without hip fracture, 9,337 lacked fall risk scores, yielding 48,692 non-hip fracture admissions with complete data.

Across all admissions, 21% returned to the hospital within 30 days. Among hip fracture admissions, the fall risk category was not significantly associated with 30-day returns (18% high risk vs. 21% low risk, p = .128). This pattern was also observed among non-hip fracture admissions (22% high risk vs. 21% low risk, p = .107). While high fall risk scores were linked to older age (mean 88 vs. 85 years, p < .001) and longer hospital stays (4.64 vs. 2.35 days, p < .001), they were not associated with increased return rates in either group.

Among high fall risk admissions, those without hip fracture had a significantly higher 30-day return rate compared to those with hip fracture (22% vs. 18%, p = .02). Table 1 compares characteristics of admissions by hip fracture and fall risk status. High fall risk admissions without hip fracture had higher rates of stroke (8.8% vs. 3.5%, p < .001), congestive heart failure (23.6% vs. 14.3%, p < .001), chronic kidney disease (9.3% vs. 7.6%, p = .039), severe anemia (17.5% vs. 14.0%, p = .004), and hypoalbuminemia (50.8% vs. 42.0%, p = .026), suggesting a greater illness burden in this subgroup.

**Table 1:** Demographics of Eligible Admissions * Comparison within hip fracture group; ** Comparison within no hip fracture group; *** Comparison between hip fracture and no hip fracture groups among those with high fall risk (score >16). Statistical tests: Chi-square tests were used for comparisons when all expected cell counts were ≥5; Fisher’s exact tests were applied when expected counts were <5. These within- and between-group comparisons were included to explore whether associations between fall risk and hospital return were specific to patients with hip fracture or generalizable across patient populations. † Lab values were available for a subset of admissions with documented fall risk scores: Hemoglobin < 12 g/dL (Anemia) • Hip fracture: n = 1,578 (Score ≤16: n = 1,149; Score >16: n = 429) • No hip fracture: n = 40,781 (Score ≤16: n = 38,320; Score >16: n = 2,461) Hemoglobin < 10 g/dL (Severe Anemia) • Hip fracture: n = 1,578 (Score ≤16: n = 1,149; Score >16: n = 429) • No hip fracture: n = 40,781 (Score ≤16: n = 38,320; Score >16: n = 2,461) Albumin < 3.0 g/dL • Hip fracture: n = 796 (Score ≤16: n = 559; Score >16: n = 237) • No hip fracture: n = 24,136 (Score ≤16: n = 22,436; Score >16: n = 1,700) Glomerular Filtration Rate (GFR) < 30 • Hip fracture: n = 2,539 (Score ≤16: n = 2,077; Score >16: n = 462) • No hip fracture: n = 48,692 (Score ≤16: n = 46,016; Score >16: n = 2,676)

	Hip Fracture			No Hip Fracture			
	N=2,539			N=48,692			
	Low Fall Risk (Score ≤16)	High Fall Risk (Score >16)	P-value*	Low Fall Risk (Score ≤16)	High Fall Risk (Score >16)	P-value**	P-value***
Characteristic n (%)	N= 2,077	N= 462		N=46,016	N=2,676		
Female	1371 (66)	328 (71)	0.04	25071 (54.5)	1425 (53.3)	0.213	< .001
Atrial Fibrillation	359 (17.3)	105 (22.7)	0.01	10606 (23)	762 (28.5)	< .001	0.011
Altered Mental Status	3 (0.1)	2 (0.1)	0.23	203 (0.4)	32 (1.2)	< .001	0.144
Coronary atherosclerosis	213 (10.3)	68 (14.7)	0.01	8182 (17.8)	415 (15.5)	0.003	0.664
Congestive Heart Failure (CHF)	158 (7.6)	66 (14.3)	< .001	8024 (17.4)	631 (23.6)	< .001	< .001
Chronic Kidney Disease (CKD) (Stage ≥ 3)	98 (4.7)	35 (7.6)	0.013	3779 (8.2)	250 (9.3)	0.039	0.222
Chronic Pulmonary Disease	136 (6.5)	44 (9.5)	0.024	4968 (10.8)	272 (10.2)	0.305	0.673
Diabetes	191 (9.2)	42 (9.1)	0.94	5424 (11.8)	304 (11.4)	0.505	0.15
Hypertension	872 (42)	199 (43.1)	0.67	18566 (40.3)	960 (35.9)	< .001	0.003
Stroke/Transient Ischemic Attack (TIA)	23 (1.1)	16 (3.5)	< .001	1603 (3.5)	236 (8.8)	< .001	< .001
Anemia (Hemoglobin <12 g/dL) †	540 (47)	203 (47.3)	0.909	16,817 (43.9)	1,257 (51.1)	< .001	0.151
Severe Anemia (Hemoglobin <10 g/dL) †	159 (13.8)	60 (14)	0.94	5,882 (15.3)	431 (17.5)	0.004	0.073
								Hypoalbuminemia (Albumin < 3.0 g/dL) †	235 (42)	120 (50.6)	0.026	8297 (63)	837 (50.8)	< .001	0.687
																Glomerular Filtration Rate (GFR) <30†	7 (0.3)	2 (0.4)	0.754	227 (0.5)	10 (0.4)	0.387	0.849

## Discussion

This study evaluated whether fall risk scores were associated with 30-day hospital returns in adults aged 75 and older hospitalized with hip fractures. While the JHFRAT is a validated tool for identifying in-hospital fall risk, our findings show it was not associated with 30-day post-discharge hospital utilization in this population. These results align with prior evidence showing that the JHFRAT performs well for assessing in-hospital fall risk but has limited application to broader outcomes like hospital return [12]. Other studies in hip fracture populations also suggest that factors such as social support, discharge disposition, and rehabilitation services may have a greater influence on hospital returns than fall risk scores alone [13].

Notably, among high fall risk admissions, those with a hip fracture had significantly lower return rates than those without. This exploratory comparison was included to determine whether the observed patterns in fall risk and return rates were specific to the hip fracture population or reflected a more generalizable trend among high fall risk patients. These comparisons help contextualize our primary findings and generate hypotheses for future research. To further explore this finding, we assessed clinical characteristics of the two groups. High fall risk admissions without hip fractures had a significantly higher prevalence of comorbidities such as congestive heart failure (23.6% vs. 14.3%) and stroke (8.8% vs. 3.5%), both of which are known to be associated with worse post-hospital outcomes [14,15].

These findings differ from prior research in surgical and cardiovascular cohorts, where higher fall risk scores, such as those from the Hendrich II model, were associated with increased 30-day readmissions following planned procedures like joint replacement and cardiac interventions [9,10]. However, a key difference is that those studies focused specifically on readmissions, whereas our outcome included a broader category of 30-day return to hospital, encompassing readmissions, observation stays, and emergency department visits without admission. Additionally, those prior studies examined patients undergoing planned procedures, such as total joint arthroplasty or cardiac interventions, where functional assessments may more directly relate to recovery and post-discharge outcomes. In contrast, our study focused on admissions hospitalized for an acute event, hip fracture, whose post-discharge course may be more heavily influenced by care setting and social factors than by in-hospital fall risk scores alone.

Our findings highlight the need for further research to better understand the factors associated with 30-day return-to-hospital rates in older adults with hip fractures. Future studies should explore frameworks that incorporate multiple dimensions in larger and more diverse populations, such as functional status, social determinants of health, and discharge care environments, to more comprehensively evaluate post-discharge risk. Longitudinal studies examining how specific rehabilitation settings or transitions of care relate to 30-day return rates could provide valuable insights. Additionally, research into interventions tailored for patients with complex needs, such as enhanced discharge planning or home-based follow-up programs, may help reduce unnecessary returns to the hospital.

Limitations

Several limitations warrant consideration when interpreting the findings of this study. First, as a retrospective analysis, causal inferences cannot be drawn, and unmeasured confounding remains possible since we did not conduct multivariable adjustment to account for covariates that were not available to us, such as discharge disposition or social factors. The dataset was derived from a single community teaching hospital, potentially limiting the generalizability of the findings to other healthcare settings with different patient demographics or care protocols. Second, the accuracy of the JHFRAT depends heavily on the training and consistency of the nursing staff responsible for the assessments. We do not know how the nurses in our hospital were trained or the inter-rater variability of the scores. Variations in training and interpretation of assessment criteria could result in discrepancies in scoring, potentially affecting the study’s results. While the data included comprehensive clinical and demographic characteristics, there were unrecorded variables, such as social support systems and specific post-discharge locations and rehabilitation protocols, that could have influenced return-to-hospital rates. Confidence intervals were not reported due to small subgroup sizes and limited return events, which would result in wide intervals unlikely to change interpretation. The application of the >16 cutoff for post-discharge prediction is a methodological limitation, as it was validated for in-hospital fall risk and may not reflect thresholds relevant to readmission risk. Finally, there is a potential selection bias in our data because admissions without documented fall risk scores were excluded from analysis. 

Despite these limitations, our data contribute valuable insight into the limited association between fall risk scores and 30-day return to hospital rates in patients aged 75 and older with hip fractures. The findings underscore the need for more comprehensive approaches that integrate clinical, functional, and social factors when evaluating post-discharge risk in older adults.

## Conclusions

Although the JHFRAT is widely used to assess in-hospital fall risk, this study found that JHFRAT scores were not significantly associated with 30-day hospital returns in older adults with hip fractures. Given the retrospective design, these results should be interpreted as exploratory associations rather than causal relationships. This suggests that fall risk at the time of hospitalization may not adequately capture the complexity of factors influencing post-discharge outcomes in this population.

These findings support the need for multifactorial approaches that consider not only clinical risk factors but also functional status, social support, and discharge planning. Broader risk models may help identify patients at greatest risk for early return to the hospital and inform targeted interventions to improve outcomes in this vulnerable population.

## Appendices

Appendix A 

**Table 2:** International Classification of Diseases, 10th Revision (ICD-10) Codes Used to Identify Hip Fracture This table lists the ICD-10-CM diagnosis codes [11] used to identify hospital admissions for hip fractures in this study. All codes represent initial encounters for acute hip fractures, including fractures of the femoral neck, pertrochanteric region, and subtrochanteric region. The 7th character “A” denotes treatment during the initial active phase of care. Codes were included regardless of laterality.

ICD-10 Code	Description
S72.001A	Fracture of unspecified part of neck of right/left femur, initial encounter
S72.002A	Fracture of unspecified part of neck of right/left femur, initial encounter
S72.009A	Fracture of unspecified part of neck of right/left femur, initial encounter
S72.101A	Pertrochanteric fracture of right/left femur, initial encounter
S72.102A	Pertrochanteric fracture of right/left femur, initial encounter
S72.109A	Pertrochanteric fracture of right/left femur, initial encounter
S72.201A	Subtrochanteric fracture of right/left femur, initial encounter
S72.202A	Subtrochanteric fracture of right/left femur, initial encounter
S72.209A	Subtrochanteric fracture of right/left femur, initial encounter
